# Interventions to support self-management in cancer pain

**DOI:** 10.1097/PR9.0000000000000690

**Published:** 2018-10-05

**Authors:** Yousuf ElMokhallalati, Matthew R. Mulvey, Michael I. Bennett

**Affiliations:** Academic Unit of Palliative Care, Leeds Institute of Health Sciences, School of Medicine, University of Leeds, Leeds, Unite Kingdom

**Keywords:** Cancer pain, Self-management, Support, Education intervention

**Key Points**

1. Lack of skills and knowledge, and misinformed attitudes toward pain and its management have been found to inhibit optimal pain control.
2. Self-management support comprises the strategies and interventions by health care professionals to improve patient's skills, knowledge, and confidence to manage their own condition effectively.
3. Tailored information provision combined with enablement strategies leads to improvements in quality of life and decreased pain intensity.
4. Effective interventions should target meaningful outcomes for patients such as self-efficacy and interference of pain with daily activities.

## 1. Introduction

### 1.1. Cancer pain

Cancer pain is one of the most frequent and distressing symptoms of malignant diseases, which has a negative impact on the quality of life for both patients and carers.^35^ A recent meta-analysis suggests a pooled prevalence rate of 51% for pain in cancer patients regardless of their disease stage and 66% in those with advanced metastatic or terminal disease.^36^ Guideline-based treatment can significantly control cancer pain and it is estimated that cancer pain may be significantly relieved in between 70% and 90% of the cases with available analgesic therapies.^10,13^ Despite this, many patients continue to experience inadequate pain management and it has been estimated that around one-third of patients do not receive pain medication proportional to their pain intensity.^11,19^ The complex, biopsychological, and subjective nature of pain makes it a difficult symptom to measure and therefore to treat.^38^ Currently, there is no agreement on which is the best instrument to measure cancer pain.^26^

Home is the preferred place of care and death for cancer patients approaching the end of life.^17,32^ However, cancer patients who receive care at home are less likely to have access to adequate analgesia compared with hospice or hospital.^10,15^ In addition, poorly controlled pain remains the main reason for patients with cancer to visit emergency departments and contact out-of-hours primary care services.^2,28^

An important factor for patients and carers managing cancer pain at home is having adequate knowledge and understanding of pain and analgesic medications.^22^ This has important influences on the quality of pain management for patients at home.^22^ Factors such as knowledge deficits, insufficient information, and misconceptions regarding pain management have been found to inhibit optimal pain control.^4,8,22^ In addition, the knowledge and attitudes of health care providers towards analgesia and supporting self-management have an important influence on effective pain management for cancer patients.^1^ Therefore, interventions that target knowledge deficits and support self-management behaviours in patients, carers, and health care professionals can improve pain and quality of life outcomes for cancer patients and their carers.^1,26^

### 1.2. Self-management

Self-management of cancer pain can be defined as “the process in which patients with cancer pain make the decision to manage their pain, enhance their self-efficacy by solving problems caused by the pain, and incorporate pain-relieving strategies into daily life, through interactions with health care professionals.”^37^ Lorig and Holman^25^ suggested 3 tasks of self-management interventions:

(1) Managing medical treatment, including self-monitoring of pain, obtaining the prescribed pain medication, and using nonpharmacologic pain management techniques.^22^
(2) Modifying and adjusting their lifestyle, employment, and behaviours to keep some amount of normalcy in life.
(3) Managing emotional and psychological consequences of illness including the use of stress-coping strategies.

Effective self-management is a continuous dynamic process that encompasses the capability to monitor one's condition and to effect the cognitive, behavioural, and emotional responses required to keep a meaningful quality of life.^5^ Patients need professional support to manage the tasks of self-management and to reach their own personal health care goals.

Self-management support has been defined as “the systematic provision of education and supportive interventions by health care staff to increase patients' skills and confidence in managing their health problems, including regular assessment of progress and problems, goal setting, and problem-solving support.”^3^ Self-management interventions are distinct from traditional education, in that they assume that the patient is active in the process.^9^ While traditional education offers disease-specific information and technical skills, self-management emphasises the application of skills such as problem-solving to one' own condition.^5^ Interventions to support self-management are more than just providing solutions to patients' problem; rather, they empower patients to solve their own problems by teaching them problem-solving skills and strategies.^26^ Interventions supporting self-management are well established for some chronic diseases such as asthma and arthritis, but they are quite nascent for cancer pain.^25,31^

## 2. Interventions to support cancer pain self-management

### 2.1. Theoretical framework

**Figure 1.**
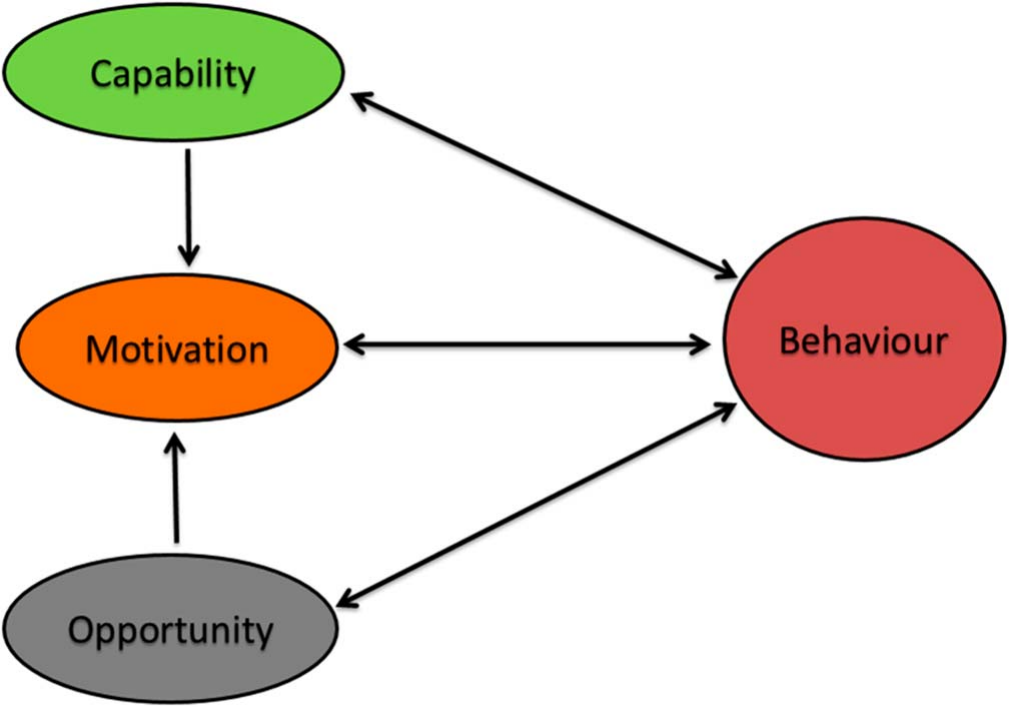
The COM-B system—a framework for understanding behaviour. Data adapted from Ref. 29.

In general, “behaviour change interventions” can be defined as coordinated sets of techniques, used together, which aim to change the health behaviours.^30^ Based on a systematic review of existing theoretical frameworks, Michie et al.^29^ have created a coherent and comprehensive framework for characterizing and designing behaviour change interventions. The model suggests that behavioural change interventions work by affecting one or more of these significant components: capability, opportunity, and motivation.^26,29^ Michie defines “capability” as an “individual's psychological and physical capacity to engage in the activity concerned,” which is dependent on having the requisite knowledge and skills.^29^ This theory suggests that knowledge and skill are potential targets for improving an individual's capability to self-manage. “Opportunity” is defined as the external factors that make enacting the behaviour possible or prompt it.^29^ Finally, “motivation” is defined as cognitive processes “that energize and direct behaviour.”^29^ The theory suggests that goal setting and shared decision-making that target “habitual processes, emotional responding, as well as analytical decision-making” may be effective strategies to increase an individual's motivation to self-manage.^29^ Michie et al.^29^ argue that it is necessary to have appropriate person-centred support to enable individuals posed with self-management challenges to enact the necessary volitional behaviour. Figure 1 (adapted from Michie et al., 2011) describes how an individual's capability “C,” opportunities “O,” and motivation “M” interact to generate behaviour “B,” which in turn influences these components. The authors describe this interaction as the “COM-B” system.^29^

Interventions to support cancer pain self-management should be based on a theoretical framework to provide directions on how to change knowledge, skills, and attitudes. However, a recent review found that only very few were based on an underlying theoretical model,^22^ which could lead to inconsistent effects of these interventions.^1,23^ A systematic review and meta-analysis by Marie et al.^27^ used the “COM-B” system to understand the mechanisms of complex interventions and evaluate their effectiveness. The meta-analysis showed that interventions using all 3 components: capability, opportunity, and motivation were efficacious and associated with a significant impact on pain intensity, whereas those that used only the 2 elements, capability and motivation, were not.^27^ Another systematic review found that many trials have been conducted without a theoretical framework for mechanism of action of self-management interventions, which could be one reason for the inconsistent effects of these interventions.^1^ To increase efficacy of the interventions and improve cancer pain outcomes, the development and design stages of the interventions require more attention.^1,23^ This can be achieved by using theory to inform interventions and including the 3 components: capability, opportunity, and motivational factors in the interventions.^1,23,27^

### 2.2. Key components

Interventions to support effective self-management for cancer pain usually include a number of core components applied in several formats such as video, audio, or written materials, which are delivered by trained health care professionals.^1^ The published evidence describing cancer pain self-management interventions is heterogeneous and varies greatly in type, delivery, content, duration, and outcome measures.^20,32^ To date, it is unclear which structure and content components lead to improvement in cancer pain outcomes.^22^ To identify the core characteristics and components of self-management interventions, numerous reviews have tried to create a taxonomy of intervention structure and components.^1,16,26,27^ For instance, Flemming et al.^16^ integrated both quantitative and qualitative systematic reviews to identify components of interventions to support self-management for cancer pain. Lovell et al.^26^ also used the behaviour change wheel as a framework to identify the key principles of self-management interventions for cancer pain. Despite that, there is still no general agreement in the literature about accepted taxonomy.^1^ Therefore, based on the work of both Flemming et al.^16^ and Lovell et al.,^26^ we have created a taxonomy that summarises the main structure and key components of existing interventions.

#### 2.2.1. Individualised and patient-centred

Pain is an individual, multifactorial experience that is usually influenced by several factors including social, psychological, and environmental.^33^ Based on the available evidence, patient-centred interventions can help improve patients' quality of life and decrease their pain intensity.^14,24,26,34^ Tailored interventions ensure that patients receive individualised education and training based on their specific needs, concerns, and gaps in knowledge combined with questions during and after the intervention to check understanding.^22,23^ Interventions should be individualised and culturally appropriate to improve knowledge and beliefs about pain and its management.^16^

#### 2.2.2. Addressing knowledge, skills, and attitudes towards pain and its management

Interventions to support cancer pain management should improve patients' and carers' knowledge and skills, and encourage positive attitudes towards pain and its management.^14,16^ These include providing information about the nature of pain, pain medications and their adverse effects, and nonpharmacological pain management techniques.^1,16^ Other strategies included support training sessions that enable patients to use numeric self-rating scales to quantify pain severity and coaching sessions that are aimed at helping patients to take their medications more regularly at the proper intervals.^26,32^ Family carers play a central role in pain management for patients with cancer.^22^ A recent systematic review by Latter et al.^23^ found that involving caregivers during face-to-face education can improve their knowledge and self-efficacy for managing pain medicines.

#### 2.2.3. The importance of an “enablement” approach

Enablement is defined as “increasing means and reducing barriers to increase capability or opportunity beyond education and training and environmental restructuring.”^29^ Enablement approach strategies are aimed at ensuring patients' engagement in their own pain management. This can be achieved mainly by improving communication with health professionals and encouraging patients and family carers to actively participate in decision-making.^27^ Different enablement approach strategies have been described, including use of a question prompt list, personalised treatment plans, provision of a telephone helpline, and instructions on how and when to contact health care providers about pain.^26^ Using an enablement approach within interventions helps patients to overcome barriers to pain management and decrease pain intensity.^16,27^

#### 2.2.4. Delivery of intervention: its format and duration

Interventions to support self-management are heterogeneous and have varied widely in format, duration, and intensity.^22^ Intervention studies were categorised according to:

(1) intervention type (face-to-face coaching sessions, individualised or group education sessions, or training sessions);
(2) materials provided (information sheets, video, audiotapes, booklet, and pain diary);
(3) intensity (single exposure or multiple exposures);
(4) duration (sessions differ greatly but most of them were between 20 and 60 minutes or different durations depending on patient needs);
(5) place of delivery (home, hospice, or outpatient clinic);
(6) delivery personnel (nurse, physician, or researcher); and
(7) recipient (patient alone or patient and carer together).

Timing the delivery of an intervention to ensure maximum patient and carer benefit is problematic, a factor that very few studies considered.^23^ Koller suggested that the optimal time for delivering the intervention is the transition from inpatient hospital settings to home.^22^

Some systematic reviews made recommendations regarding the format of interventions but the optimal timing, provider, and duration remain unclear.^7,14,16,22^ Delivering face-to-face coaching or education session including suitable materials and follow-up can be effective in reducing cognitive barriers and improving pain management for patients and family carers.^22,23^ Cummings et al.^14^ suggested that interventions with higher educational dose (equal to or greater than 2 hours in one setting, or equal or greater to 4 teaching sessions) can improve pain management knowledge, skills, and attitudes. However, 2 other meta-analyses suggested that effect size was independent of dose.^7,18^ Until recently, standardised approaches to delivery of self-management support interventions have not been described in the literature.^22^ Effective educational materials should be available online and then it can be adapted to suit different settings and patient groups.^26^

#### 2.2.5. The importance of health care professionals' educating and training

In addition to assessment, diagnosis, and treatment, clinicians should be able to educate their patients on managing their pain.^28^ However, too few health professionals have received education and training on how to educate and support patients to manage their conditions.^21,28^ To improve intervention effectiveness, efficient and relevant education and training should be provided to health professionals to improve their skills and confidence in delivering the intervention.^15^

### 2.3. Cancer pain outcomes

The evidence of effectiveness of these self-management support interventions has been based on improvements in pain intensity using numerical rating scales, although a range of secondary outcomes have also been measured.^1,22^ To date, there is no consensus on how best to measure cancer pain outcomes that matter to patients.^7,26,32^ Primary outcomes of recent studies have shifted from improving knowledge and attitude to a clinically significant change in pain intensity.^1,22^ A systematic review and meta-analysis of 15 randomized controlled trials found that the effects of self-management interventions on pain intensity and interference of pain with daily activities were small to moderate.^27^ Another recent systematic review of 26 randomized controlled trials found that less than one-third of the included interventions improved pain intensity and significantly changed pain interference.^32^ In the same systematic review, there was a significant improvement in pain knowledge, medication adherence, and self-efficacy, suggesting these may be potential modifiable targets for self-management interventions.^32^

The majority of studies measured pain intensity; however, there was a lot of heterogeneity in the methods and tools to assess and report pain intensity, eg, 0 to 10 or 0 to 100 Numeric Rating Scale, 0 to 10 mm or 0 to 100 mm horizontal visual analogue scale, 4-point Likert scale, or a combination of different scales.^7,22^. Different methods were reported for summarising pain intensity data, such as average pain intensity or worst pain intensity, within different time frames (eg, now, the last day, last week, or not specified).^22^ Although recent systematic reviews included pain interference with daily life as a primary outcome, most previous interventions did not measure this concept.^27,32^

Other authors have suggested that self-management interventions should focus on improving the quality of life and helping patients to achieve a balance between pain and adverse effects of analgesia.^6^ Although monitoring changes in pain intensity may be a meaningful outcome for health care professionals, it can have less meaning for patients, for whom maintaining relationships and achieving usual daily activities are often the key goals associated with a “good” quality of life.^12^ Self-management interventions for cancer pain should assess more holistic outcomes such as interference with functioning, and effects on general quality of life.^6,27,38^

### 2.4. Integrating self-management support for cancer pain into routine clinical practice

Interventions to support cancer pain self-management have the potential to improve patient outcomes. However, the effects of these interventions are often short term and limited in their ability to reach all patients with cancer pain.^21^ Numerous studies suggest that health professionals should integrate interventions to support self-management into usual clinical practice but implementation is still variable across settings.^7,16,22,26^

To implement a sustainable intervention, clinicians should consider the available resources and the standard care in their settings.^34^ Moreover, health professionals must be involved in designing the interventions to make them suitable and compatible with their clinical practice.^23^ Pain assessment, timely pain reassessment, and identifying barriers to effective pain management should be part of their routine practice.^1^ In addition, ongoing support and advice should be provided to patients based on their needs and the severity of pain.^34^ Effective communication between members of the multidisciplinary team can enhance intervention fidelity and reduce care fragmentation.^26^ Examples of simple practical activities and tips that most health care professionals can incorporate into their practice are provided in Table 1.

**Table 1:**
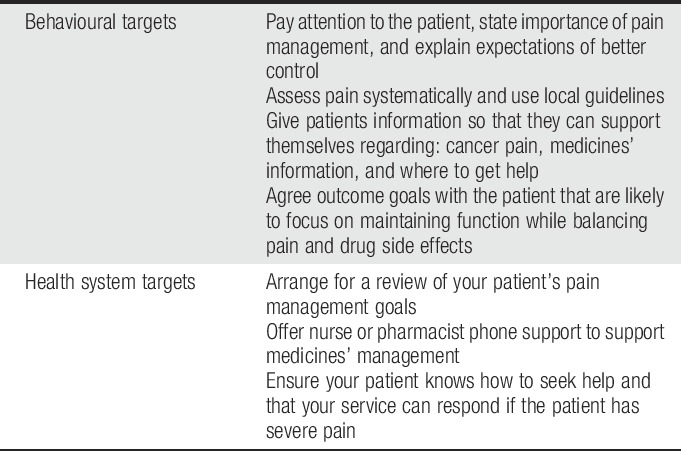
Practical tips for clinicians to support self-management of cancer pain.

## 3. Conclusion

To provide long-term support for a wider population of cancer patients with pain, clinicians should integrate evidence-based activities to support self-management into routine clinical practice.

## Disclosures

The authors have no conflict of interest to declare.
